# Supplementary figures and images for: Evolutionary insights into provirus-encoded CRISPR-Cas systems in halophilic archaea
Source: Microlife. 2025 Oct 22;6:uqaf033. doi: 10.1093/femsml/uqaf033 (PMC12596717; doi:10.1093/femsml/uqaf033)

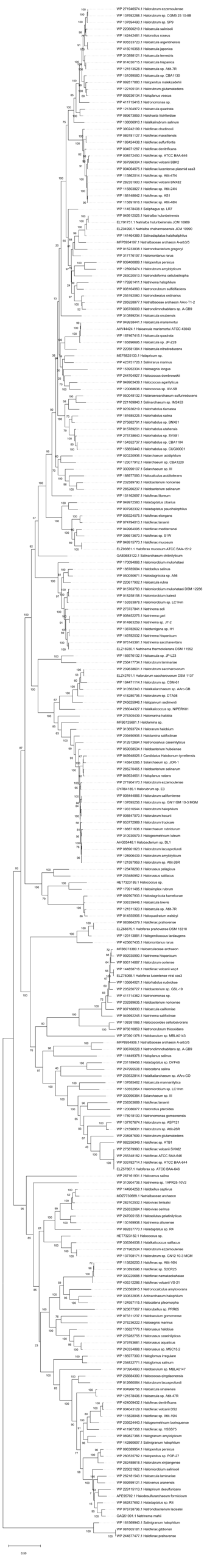

Supplement: uqaf033_Supplemental_Files [file uqaf033_supplemental_files.zip › supplementary_data_1(trees)/supplementary_data_1/trees/cas3_final_tree.pdf]

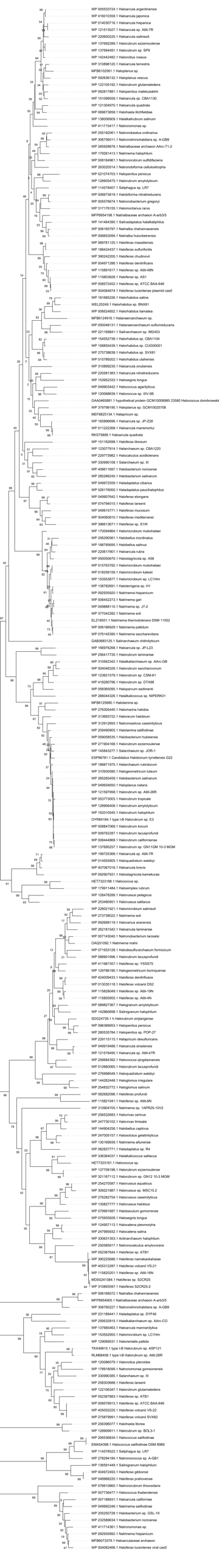

Supplement: uqaf033_Supplemental_Files [file uqaf033_supplemental_files.zip › supplementary_data_1(trees)/supplementary_data_1/trees/cas5_final_tree.pdf]

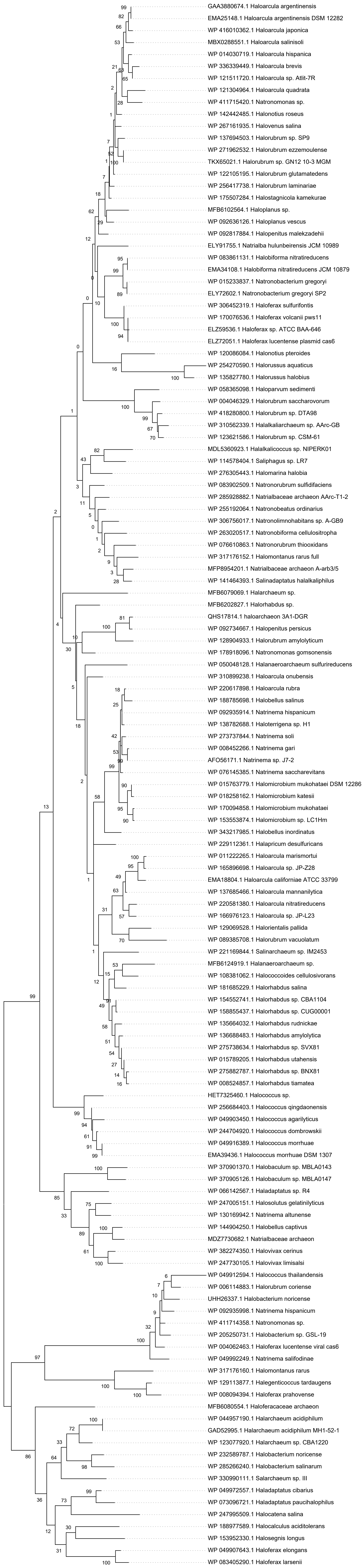

0.20

Supplement: uqaf033_Supplemental_Files [file uqaf033_supplemental_files.zip › supplementary_data_1(trees)/supplementary_data_1/trees/cas6_final_tree.pdf]

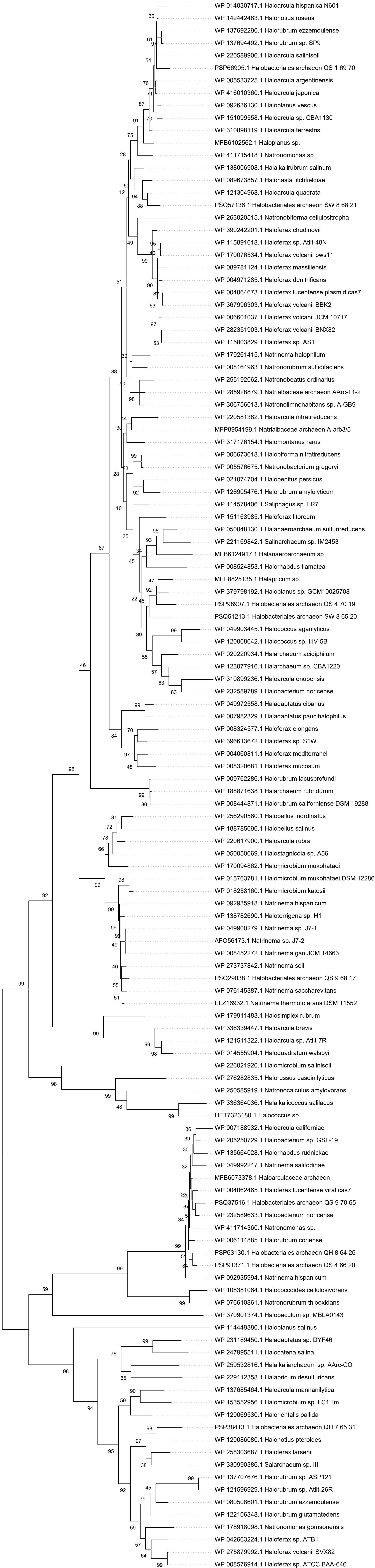

0.20

Supplement: uqaf033_Supplemental_Files [file uqaf033_supplemental_files.zip › supplementary_data_1(trees)/supplementary_data_1/trees/cas7_final_tree.pdf]

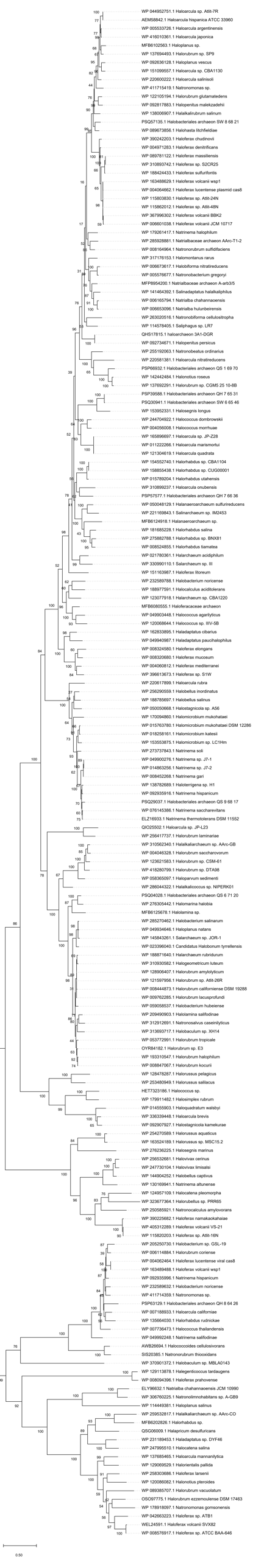

0.50

Supplement: uqaf033_Supplemental_Files [file uqaf033_supplemental_files.zip › supplementary_data_1(trees)/supplementary_data_1/trees/cas8_final_tree.pdf]

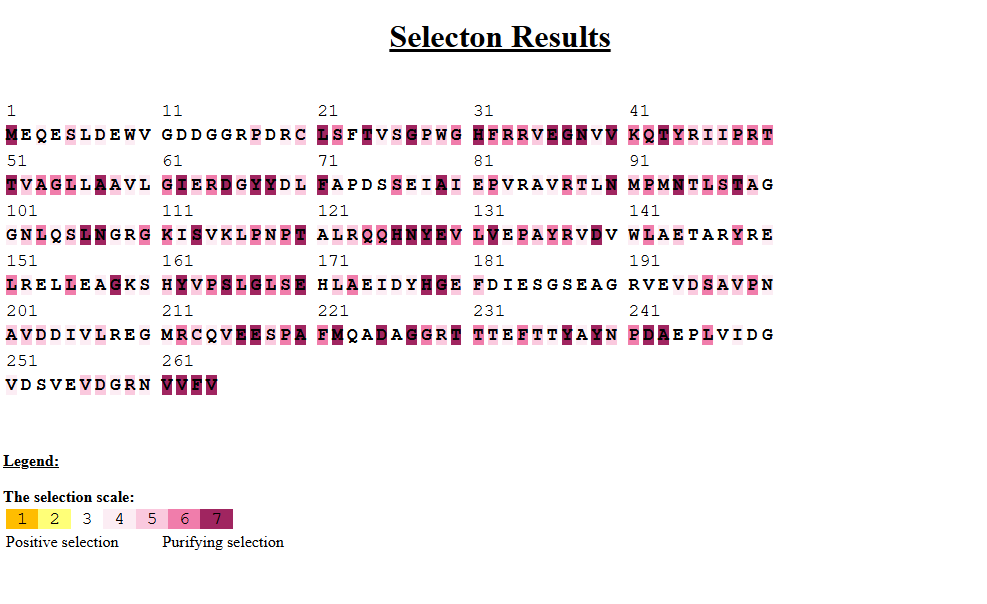

Supplement: uqaf033_Supplemental_Files [file uqaf033_supplemental_files.zip › supplementary_data_2(selecton)/supplementary_data_2/selecton color tables/cas5_plasmid_resutls_selecton_final.png]

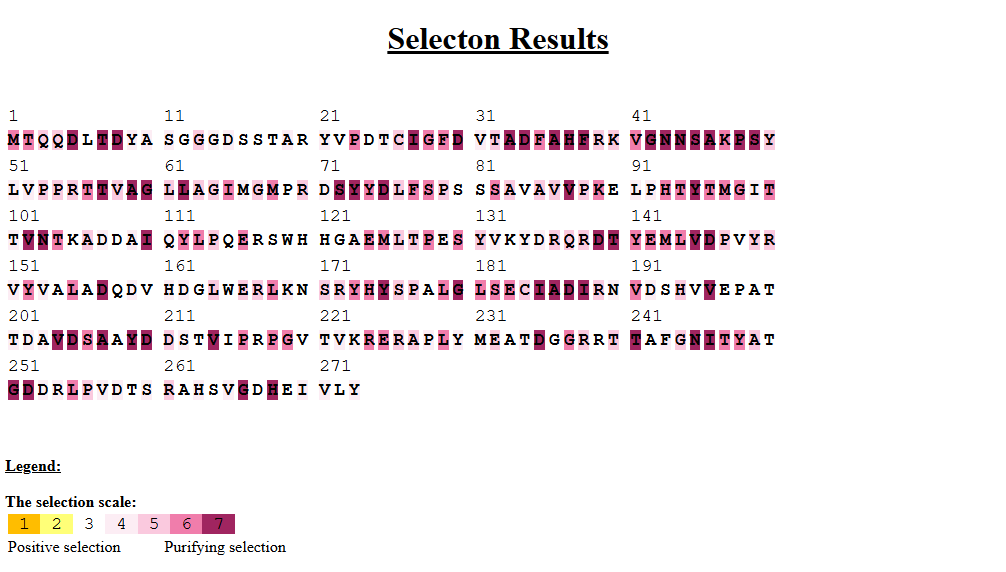

Supplement: uqaf033_Supplemental_Files [file uqaf033_supplemental_files.zip › supplementary_data_2(selecton)/supplementary_data_2/selecton color tables/cas5_viral_final_selecton.png]

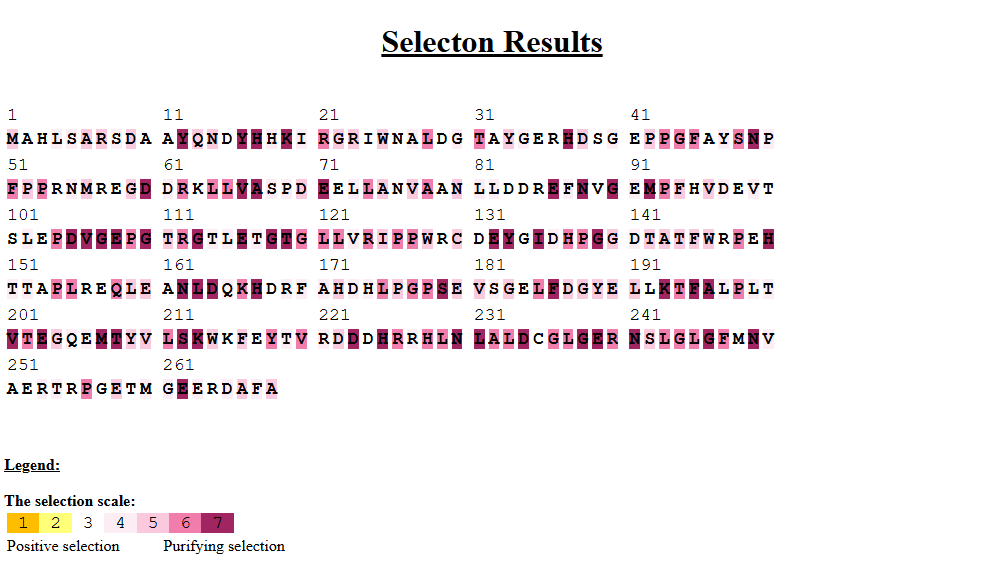

Supplement: uqaf033_Supplemental_Files [file uqaf033_supplemental_files.zip › supplementary_data_2(selecton)/supplementary_data_2/selecton color tables/cas6_plasmid_results_selecton_final.png]

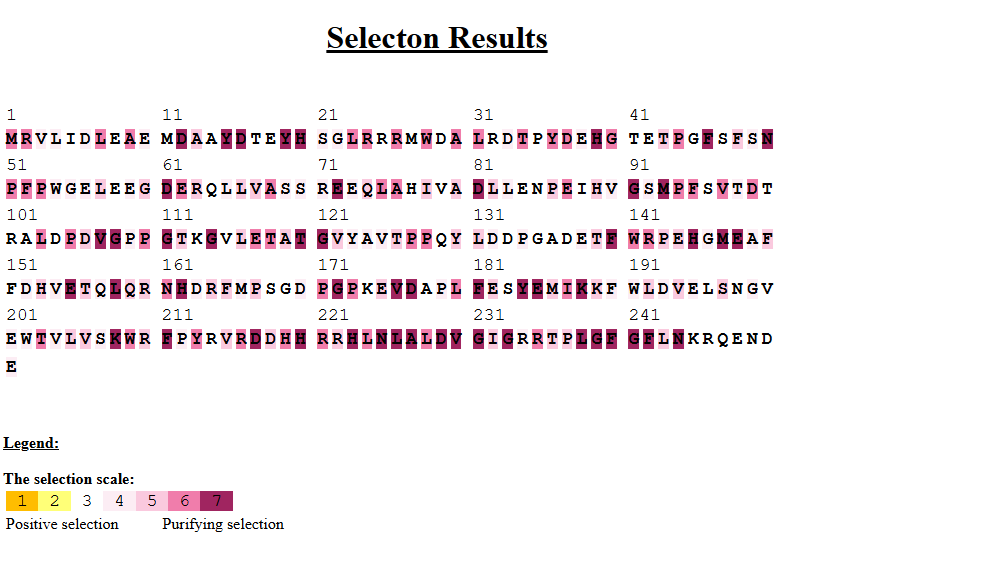

Supplement: uqaf033_Supplemental_Files [file uqaf033_supplemental_files.zip › supplementary_data_2(selecton)/supplementary_data_2/selecton color tables/cas6_viral_selecton_final.png]

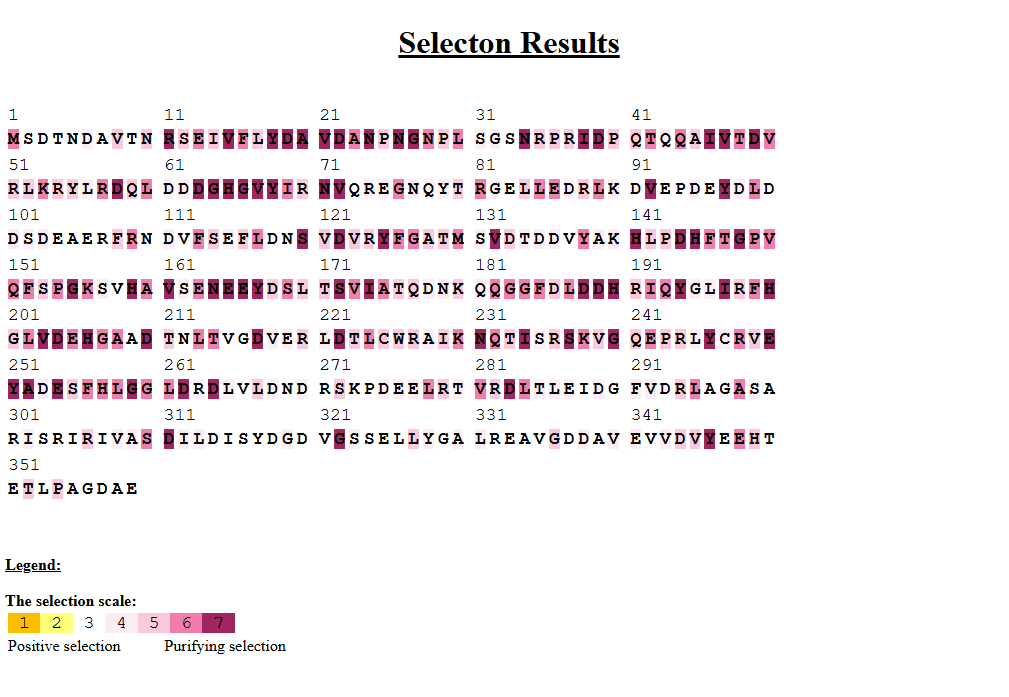

Supplement: uqaf033_Supplemental_Files [file uqaf033_supplemental_files.zip › supplementary_data_2(selecton)/supplementary_data_2/selecton color tables/cas7_plasmid_results_selecton_final.png]

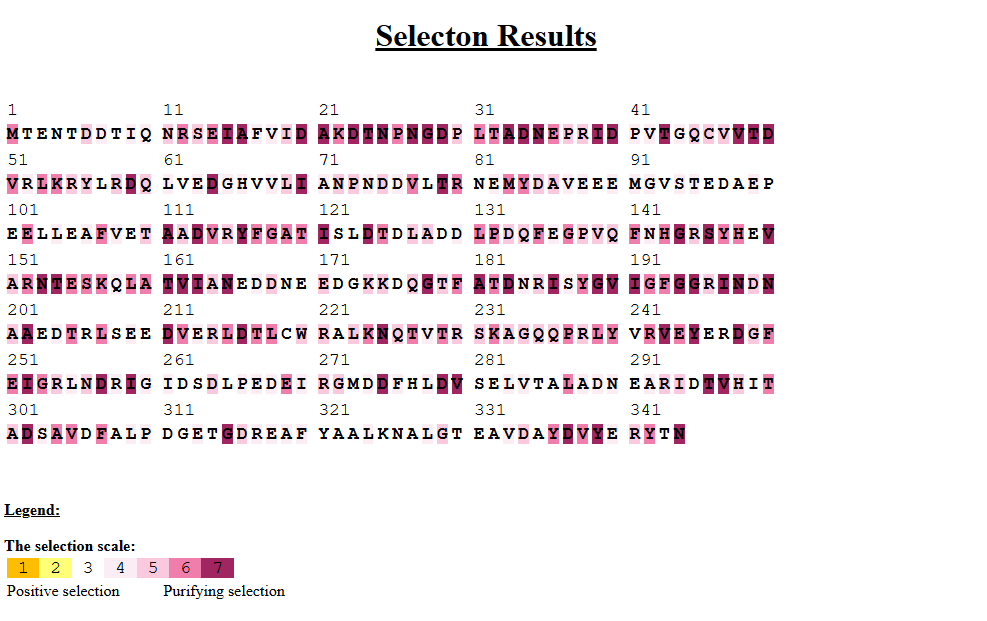

Supplement: uqaf033_Supplemental_Files [file uqaf033_supplemental_files.zip › supplementary_data_2(selecton)/supplementary_data_2/selecton color tables/cas7_viral_final_selecton.png]

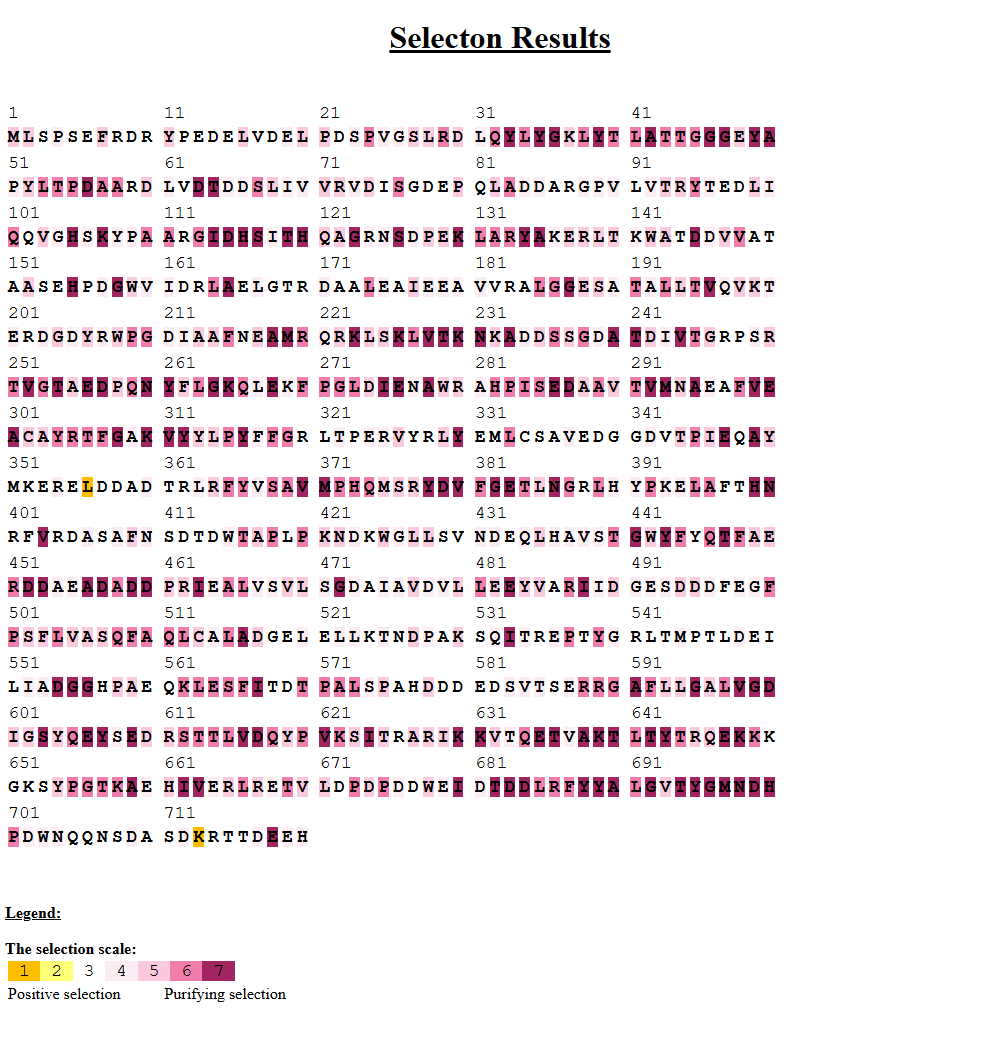

Supplement: uqaf033_Supplemental_Files [file uqaf033_supplemental_files.zip › supplementary_data_2(selecton)/supplementary_data_2/selecton color tables/cas8_plasmid_resutls_selecton_final.png]

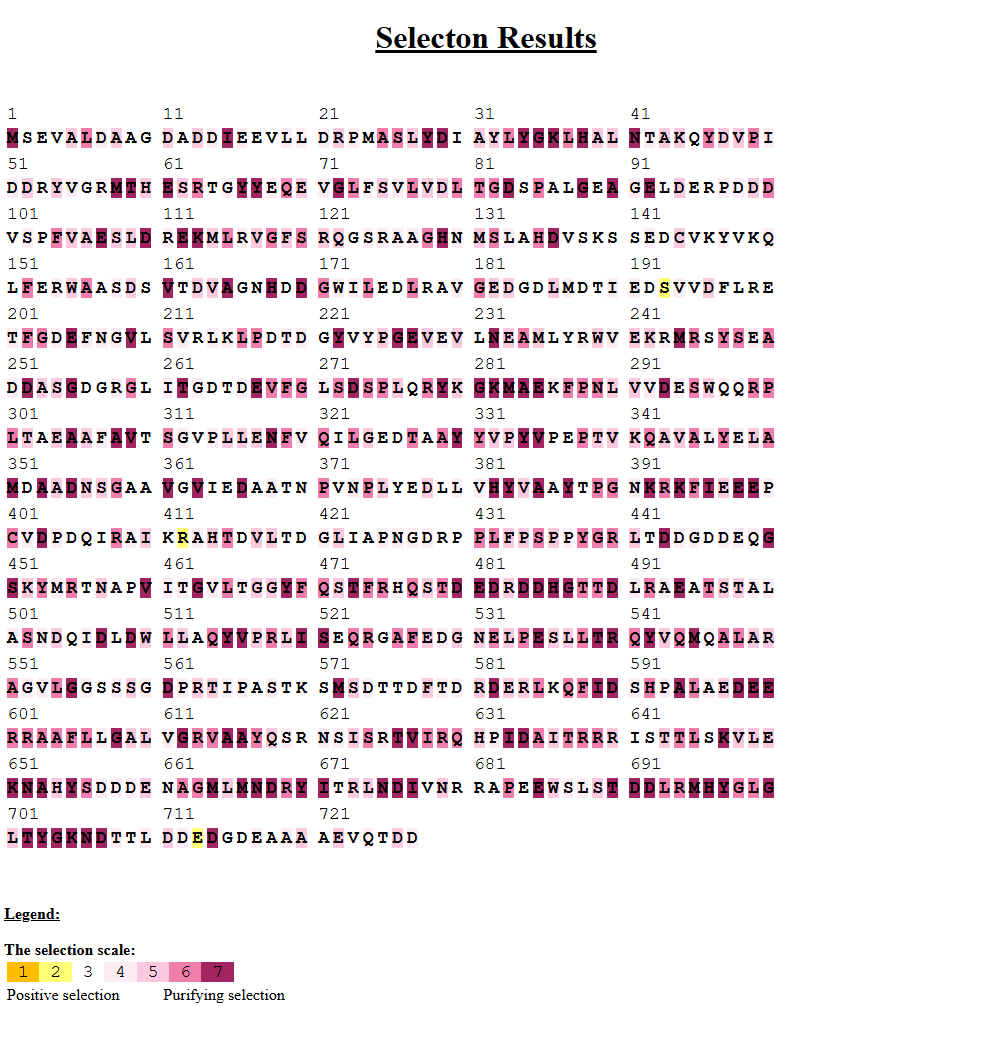

Supplement: uqaf033_Supplemental_Files [file uqaf033_supplemental_files.zip › supplementary_data_2(selecton)/supplementary_data_2/selecton color tables/cas8_viral_selecton_final.png]

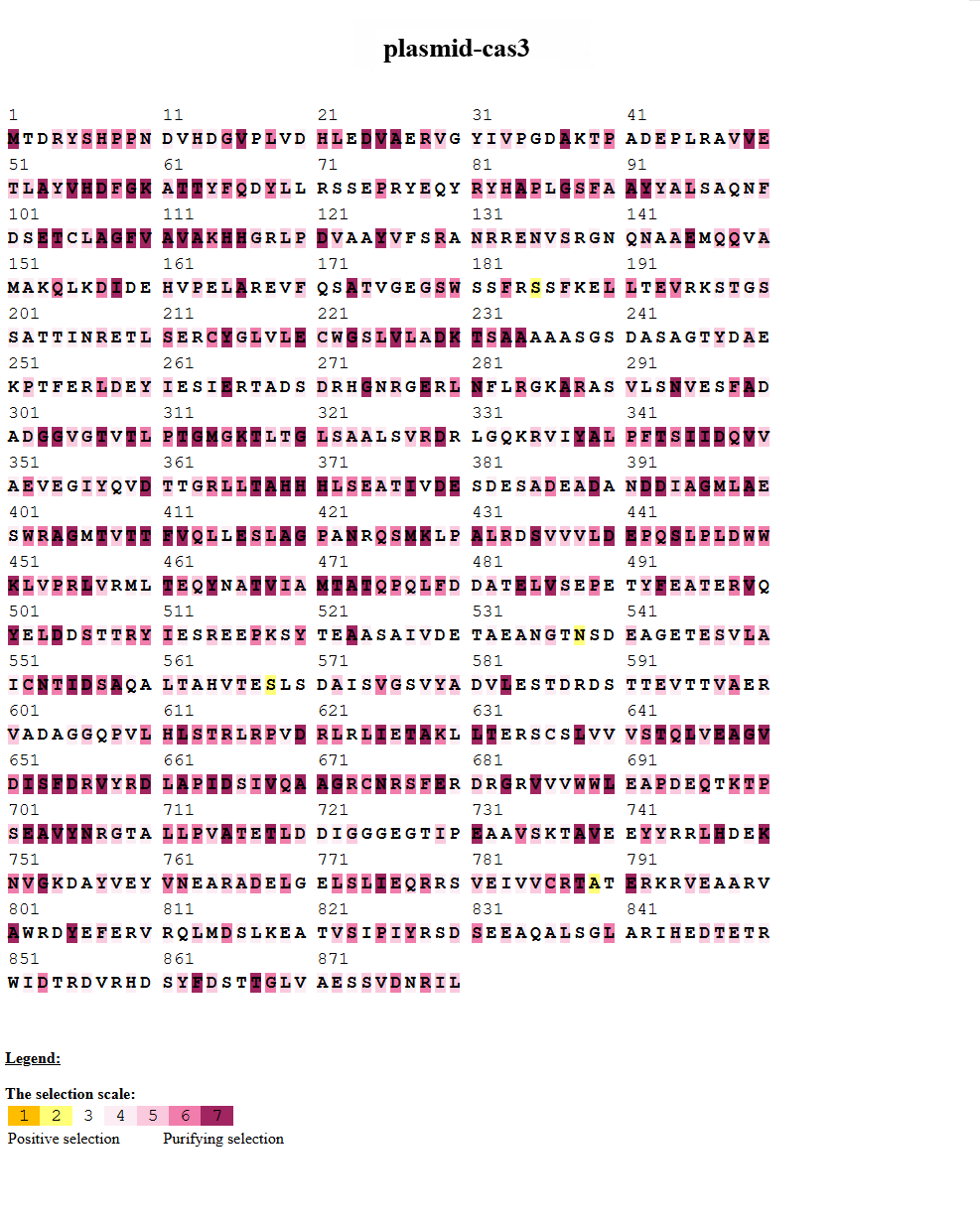

Supplement: uqaf033_Supplemental_Files [file uqaf033_supplemental_files.zip › supplementary_data_2(selecton)/supplementary_data_2/selecton color tables/plasmid_cas3.png]

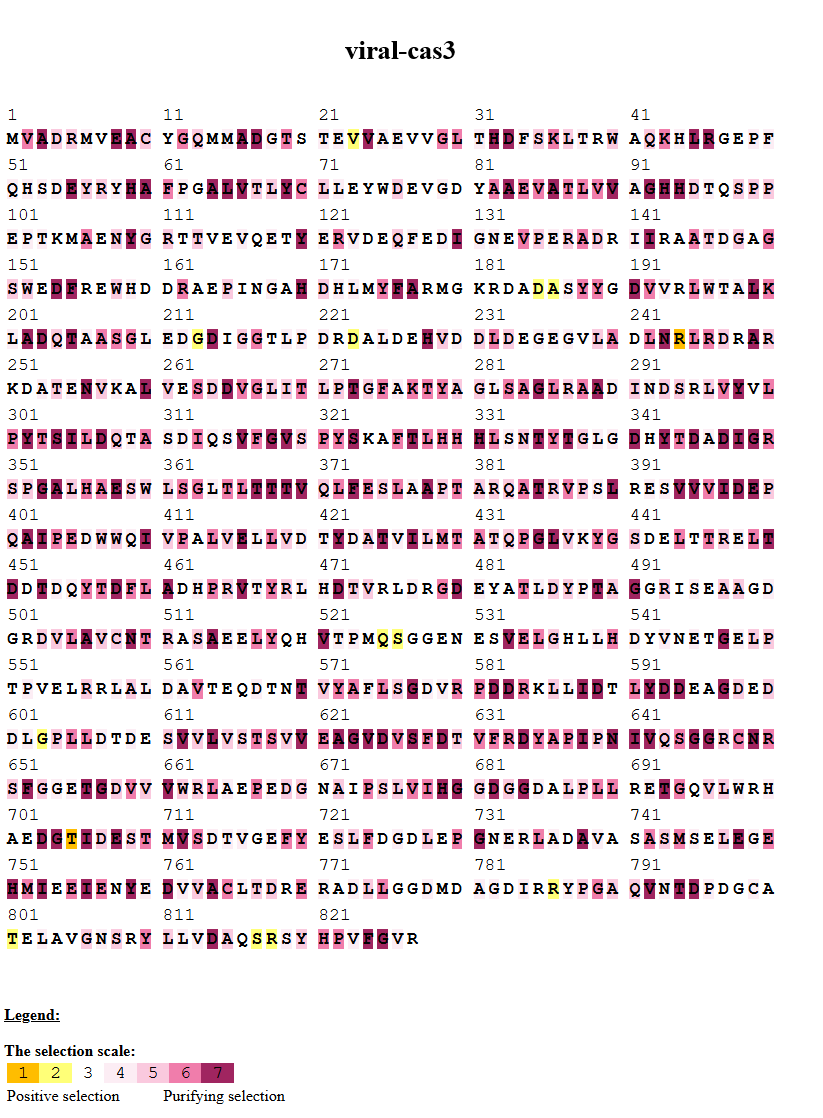

Supplement: uqaf033_Supplemental_Files [file uqaf033_supplemental_files.zip › supplementary_data_2(selecton)/supplementary_data_2/selecton color tables/viral_cas3.png]
